# A scoping review of integrated yoga and psychological approaches for the treatment of eating disorders

**DOI:** 10.1186/s40337-023-00845-5

**Published:** 2023-09-08

**Authors:** Jennifer O’Brien, Subhadra Evans, Shane McIver, Melissa O’Shea

**Affiliations:** Deakin University, Melbourne, Australia

**Keywords:** Eating disorders, Yoga, Psychological approaches, Yoga programs, Complementary treatment approaches

## Abstract

**Background:**

Eating disorders are serious mental health conditions that significantly impact the social and economic burden of mental ill health in Australia. Best practice treatment for eating disorders includes a multi-axial approach, including medical, psychiatric, and psychological approaches. More recently, complementary and alternative therapy approaches, such as yoga, are used to support eating disorder recovery.

**Methods:**

This scoping review identified and examined current research exploring the use of yoga alongside psychological approaches for the treatment and management of eating disorders across the lifespan.

**Results:**

Results highlighted the lack of available research, with only four studies identified. Three of these studies piloted programs and identified promising results with a reduction of eating disorder symptomatology. However, these results remain tentative due to methodological limitations and the overall lack of available evidence. In the future, researchers are encouraged to clearly articulate the theoretical concepts that underpin their yoga programs and focus on adequately powered and designed trials, such as RCTs, to accurately compare treatment effects between interventions combining yoga with psychological interventions and standard psychological treatment. Qualitative enquiry is also recommended to provide further insights regarding what makes interventions successful.

**Conclusions:**

Current evidence suggests further guidance and pragmatic recommendations to guide researchers and clinicians alike are required, ultimately improving outcomes for people experiencing an eating disorder across the lifespan.

## Abstract (plain language summary)

Yoga is commonly used as an additional therapy in eating disorder services. This research review aimed to identify studies that reported on how yoga was added to psychology approaches in eating disorder services. Only four studies have been published where yoga was used with psychological approaches for the treatment of eating disorders. The four studies showed yoga was overall a helpful addition. Consistent with various yoga and psychological approaches, there were differences in how each study incorporated yoga into treatment and their rationale for its inclusion. This study highlights the gaps regarding what we understand about how yoga can be a helpful addition to psychological approaches and makes recommendations for how studies in the future should explain and report the use of yoga.

## Background

Eating disorders are typically characterised by an unhealthy preoccupation with eating, exercise, body weight or shape and behaviours such as restricting food, excessive exercise, binge eating and purging [21, 51]. Individuals with eating disorders also demonstrate low recovery rates [22, 26] and high relapse rates [24], contributing significantly to the social and economic burden of mental ill health in Australia. With an estimated 4–16% of the Australian population affected across their lifespan, eating disorders represent a major public health concern [22, 23, 47].

Best practice treatment for eating disorders includes a multi-axial approach, including medical, psychiatric, and psychological approaches [22]. Research suggests numerous psychological approaches such as cognitive behavioural therapy (CBT), dialectical behavioural therapy (DBT), and family-based psychotherapies [1] can help with recovery. Although these approaches treat cognitive and emotional symptoms characteristic of eating disorders, some treatment gaps potentially remain. For example, the experiential nature of somatic or bodywork is not always incorporated or considered as part of a central focus. Furthermore, many people with eating disorders find treatment challenging, with high rates of dropout and disengagement [28]. As such, complementary and alternative therapy approaches, such as yoga, are being used in various therapeutic settings in the UK, Australia, and the US to expand treatment options and improve engagement [4].

Yoga involves breathing practices, physical poses which calms the nervous system, and meditation which often includes kindness towards oneself [17]. Research suggests yoga has positive effects on numerous psychological, biological, and social factors that correlate to factors often contributing to the development of eating disorders. For example, yoga has been found to improve psychological factors such as improved body responsiveness and awareness [16], interoception and embodiment [12, 36, 48], mindfulness, self-compassion, self-efficacy [29] body satisfaction, body appreciation and positive body image [11, 30, 37, 38]. Yoga has also been found to improve sleep [50] and lower anxiety [14] along with promoting one’s overall physical health and well-being including enhancing nervous system function [13]. Further, yoga can improve social factors by increasing the opportunity for new social connections and sense of belonging through community classes [27]. Borden and Cook-Cottone [4] conducted a meta-analysis and systematic review to assess the effects of yoga on eating disorder symptoms. This review identified yoga as having a small significant effect on reducing global eating disorder psychopathology and body image concerns and a medium significant effect on reducing binge eating and bulimia compared to control groups. Based on current evidence, they further concluded that at this stage, yoga could not be recommended as a stand-alone treatment for eating disorders but can add value in combination with other approaches.

Within the range of hypothesised psychological mechanisms linking yoga-based interventions to improvements in eating disorders symptoms, embodiment has received significant focus. Embodiment has been defined as "the lived experience of engagement of the body with the world" [46]. Several studies have explored the theoretical connection among yoga, embodiment and eating disorders [12, 43, 45], suggesting that yoga facilitates embodiment awareness which in turn supports eating disorder recovery. Specifically, it is argued as yoga facilitates a shift from a negative sense of embodiment towards more positive self-awareness and experiences. This might improve body image and wellbeing and potentially reduce eating disorder symptomatology [43].

A recently published mapping review examined how yoga might complement evidence-based psychological therapies for adults with various mental disorders, including eating disorders [40]. A single study exploring yoga alongside psychological treatment for eating disorders [8] was identified among 13 studies overall. It was a pilot study of a manualised group treatment approach incorporating yoga with DBT, CBT, and dissonance-based interventions for the treatment of anorexia nervosa (AN) and bulimia nervosa (BN). The study concluded that incorporating yoga with existing evidence-based psychological treatments had the potential to relieve eating disorder symptoms further. Notably, there is currently no examination of yoga used alongside psychological therapies for eating disorders across the lifespan. Borden and Cook-Cottone’s [4] meta-analysis and systematic review focussed on yoga as a stand-alone treatment for eating disorders and assessed efficacy outcomes, while the O’Shea et al. [40] review omitted potential studies of children and teenagers. Considering eating disorders commonly occur in late childhood and adolescence and that the treatment approaches between adolescence and adulthood are not overlapping, it is also important to review yoga alongside psychological approaches across the lifespan. The O’Shea et al. [40] review was also limited to Level I and Level II evidence-based psychological treatment approaches, therefore omitting potential studies where yoga was used with approaches such as group work or counselling.

As such, this study applied scoping review methods [3, 35, 49] to identify and review current research that has explored the use of yoga alongside psychological approaches to address this knowledge gap. Scoping reviews are useful for identifying knowledge gaps, examining a general body of literature on a particular topic, and clarifying specific concepts [35]. It was anticipated this review would serve to further our current understanding of how yoga has been used in practice, whether it complements or enhances current treatments, and in what ways.

Subsequently, a broad research question was proposed:

1. What is the scope and characteristics of the existing evidence when yoga is integrated alongside psychological approaches in the treatment of eating disorders?

## Methods

This scoping review followed Arksey and O'Malley's [3] methodological framework which has been advanced by Levac et al. [31] and Peters et al. [44]. As per the framework’s recommendations, five iterative stages were followed, (1) identifying the research questions; (2) searching for studies; (3) selecting the relevant studies (4) charting the data; and (5) collating, summarising, and reporting. Data extraction and reporting steps adopted the PRISMA extension for scoping reviews to ensure that data extraction adhered to the Preferred Reporting Items for Systematic Reviews and Meta-Analyses Extension for Scoping Reviews (PRISMA-ScR) [34, 42]. A suitable research team with expert knowledge in eating disorders, yoga and methodology knowledge and expertise was assembled to ensure the successful completion and rigour of this review.

### Stage 1: Identification of research questions

The research question was developed through discussions with all members of the research team and was based on *the Population-Intervention-Comparisons-Outcomes-Study* design (PICOS) mnemonic and designed to identify the extant literature examining interventions of integrated yoga and psychological approaches for treating eating disorders. The population was any person diagnosed with an eating disorder using any recognised diagnostic criteria and/or a standardised diagnostic assessment/tool and engaged in psychological interventions/treatment as part of their eating disorder treatment. As this was a scoping review there were no comparisons or outcome variables specified for inclusion.

### Stage 2: Search strategy

Search terms for the population were developed from the eating disorder diagnoses published in the Diagnostic and Statistical Manual (DSMV) [1]. There were no limitations on eating disorder diagnosis, age, or gender. Search terms for interventions included both yoga and psychological approaches. Inclusion criteria were any published studies that described a cohort trial, or protocol thereof, where yoga was delivered alongside or incorporated into a psychological approach to relieve symptomatology or improving functioning for the population group. Exclusion criteria were any studies that did not describe both a yoga and psychological approach. It was necessary to define what a yoga intervention was for the purposes of this study. Any style of yoga was accepted but only those studies that described the following components of yoga were included within this review: physical postures (*asana*), breath regulation/exercises (*pranayama*), and relaxation or meditation (*dhyana*), typical of hatha yoga, being the most common style of yoga in the western context [6]. We adopted Australia’s current published best practice guidelines [2] cross-referenced with the UK’s National Institute for Health and Care Excellence (NICE) [39] guidelines and developed search terms describing Level I and II evidenced psychological therapy interventions for eating disorders (see Table 1). Additional terms such as counselling, group therapy, psychological therapy, talk therapy, therapy and standard medical treatment were also included to capture other psychological approaches. Review papers were also excluded.

**Table 1:** Evidence based psychological interventions (Level I and Level II) for eating disorders

Eating disorder	Age	Level I	Level II
Bulimia Nervosa (BN)	Over 18	CBT-ED	CBT-ED
		IPT	Interpersonal Therapy
		Psychoeducation	DBT
			Bibliotherapy
			IPT
			MBSR
			Psychoeducation
	Under 18	FT-BN	
Binge Eating Disorder (BED)	Over 18	Guided self-help program	Interpersonal Therapy
		Group CBT-ED	DBT
		CBT-ED	Mindfulness Therapy
	Under 18	Guided self-help program	
		Group CBT-ED	
		CBT-ED	
		FT-BED	
Anorexia Nervosa (AN)	Over 18	Cognitive Behavioural Therapy-(CBT-ED)	Eating-disorder-focused focal psychodynamic therapy (FPT)
		Maudsley Anorexia Nervosa Treatment for adults (MANTRA)	Motivational interviewing for eating disorders
			Interpersonal Psychotherapy
		Specialist Supportive Clinical Management (SSCM)	Cognitive Analytical Therapy
			Focal Psychoanalytic
			Psychodynamic Therapy

Adapted from [2] and [37]

The search was conducted in April 2022 through electronic database searches of CINAHL, Embase, MEDLINE, and PsycINFO. Hand searching including reviewing reference lists of included studies was also undertaken in an attempt to identify additional studies not identified through this search, but no further studies were identified. Table 2 below describes the final search strategy and key search terms identified.

**Table 2:** Search Strategy

*Databases searched *via* EBSCO host 1900–2022:* MEDLINE PsycINFO CINAHL Embase
*Search terms:* 1. AB yoga AND 2. AB ("anorexia nervosa" OR anorexia OR anorexic OR "bulimia nervosa" OR bulimic OR bulimia OR "binge eating disorder*" OR "eating disorder*" OR "other specified feeding or eating disorder*" OR OSFED OR "atypical anorexia nervosa" OR AAN OR "avoidant restrictive food intake disorder*" OR ARFID) OR AB ( "anorexia nervosa" OR anorexia OR anorexic OR "bulimia nervosa" OR bulimic OR bulimia OR "binge eating disorder*" OR "eating disorder*" OR "other specified feeding or eating disorder*" OR OSFED OR "atypical anorexia nervosa" OR AAN OR "avoidant restrictive food intake disorder*" OR ARFID) AND 3. AB (cbt OR “cognitive behavioural” or "cognitive behaviour therap*" OR "cbt eating disorder focused" OR "online CBT" OR "online cognitive behavioural therap*" OR "psychodynamic therap*" OR psychoeducation* OR "dialectical behavior therap*" OR dbt OR IPT OR "interpersonal therap*" OR "family based therap*" OR "family intervention*" OR FI OR FBT OR MBSR OR "mindfulness- based stress reduction" OR "acceptance and commitment therap*" OR ACT OR "emotion- focused therap*" OR EFT) OR AB ( ( cbt OR "cognitive behaviour therap*" OR "cbt eating disorder focused" OR "online CBT" OR "online cognitive behavioural therap*" OR "psychodynamic therap*" OR psychoeducation* OR "dialectical behaviour therap*" OR dbt OR IPT OR "interpersonal therap*" OR "family based therap*" OR "family intervention*" OR FI OR FBT OR MBSR OR "mindfulness-based stress reduction" OR "acceptance and commitment therap*" OR ACT OR "emotion- focused therap*" OR EFT OR “counselling” OR “group therapy” OR “psychological therapy” OR “talk therapy” OR therapy OR “standard medical treatment” OR “standard medical care” or “treatment as usual” OR outpatient* OR treatment OR psychotherapy)
*Limits:* No limits implemented in search strategy
*PICOS:*
*Population:* Any age, any gender, diagnosed with an eating disorder using any recognised diagnostic criteria and/or a standardised diagnostic assessment/tool by a clinician
*Intervention:* Studies which examined hatha yoga as an integrated or adjunct part of a psychological approach
*Comparison:* No restrictions on comparison
*Outcomes:* No restrictions on outcome variables
*Study types:* Any study which described a cohort trial, or protocol thereof, with the relevant interventions and populations
*Exclusion:* 1. Studies that did not describe an intervention (i.e. review paper) or did not assess a cohort (i.e. case studies)

### Stage 3: Study selection

Duplicates were removed from the final search and titles and abstracts of all studies were screened according to the inclusion and exclusion criteria. A full-text review of studies that met the criteria was undertaken (Table 3).

**Table 3:** The scope of the evidence base for integrated yoga and psychological approaches for the treatment of eating disorders

Author	Interventions	Study aim	Participants	Study design	Measures	Outcome	Limitations	Quality	Theoretical framework
Cook-Cottone et al. [8] USA	Eight weekly two-hour group sessions of the attunement in mind, body, and relationship (AMBR) program *Psychological Intervention*: Incorporates CBT and DBT with dissonance-induction content *Yoga Intervention:*50-min of body-focused yoga and 15-min of meditation/ relaxation practices were incorporated into the weekly group sessions of AMBR. Participants were instructed on breathing and self-talk during postures, which increased incrementally in difficulty over the eight weeks	To describe the theoretical foundations and examine preliminary outcomes for treating eating disorders with the AMBR program	29 women diagnosed with anorexia or bulimia nervosa engaged in an eating disorder treatment clinic (ages 14–30 years; mean age = 20 years) across one six-session group and four 8-session groups N = 24 completing Aged between 20 and 25 years (Mean 20 years) Attrition: 44 participants signed up for the program with 29 (66%) completing it	Single group pilot study with pre- post data	Three subscales from the Eating Disorder Inventory-2 (EDI-2; Garner, 1991): Drive for Thinness (DT), Body Dissatisfaction (BD); Bulimia (BU)	Significant reductions were observed in participants’ DT and BD scores after the program No significant differences were observed in BU scores	Preliminary support for an integrative mind body treatment encompassing aspects of CBT, DBT and relational theory in the treatment of eating disorders Further research is warranted for empirical validation of positive findings using RCT design and more robust outcomes measures, as well as qualitative data collection to assess participant experiences and feedback	*Level 3* Non-randomized controlled cohort/follow-up study	Integration of yoga and psychological therapy based on ‘Theoretical Attunement Framework’. Weekly themes were taken from the Theoretical Attunement Framework
Diers et al. [18] USA	Eight weekly 90 min group sessions of the Yoga Body Image (YBI) program *Psychological Intervention*: 45-min group discussion after yoga practice to allow discussion about class theme aimed to aid in the participant’s verbal processing of their experience in sensation, emotion and overall experience. Goal setting also used *Yoga intervention:* 45 min of therapeutic yoga that incorporated aspects of hatha, vinyasa, and viniyoga. Each session focused on a different theme related to body image such as body awareness and appreciation, free movement, non-judgement, emotional connection, breathing, body-mind capabilities, resiliency and neutrality. Yoga postures (asanas) breath technique (pranayama) and guided imagery selected to support theme	To examine the programs acceptability and perceived impact on body image as reported by participants before and after the program	91 participants expressed interest in the study and took first class of YBI 67 participants completed both pre and post questionnaires N = 67 66 = female 1 = male No age range given. No specific ED diagnosis given	Pilot Feasibility study	Survey data was used to quantitatively examine changes in body image concern scores before and after program Open ended survey questions used to understand observed changes in body image scores and as a phenomenological exploration of the mechanisms through which yoga might influence body image concerns	Statistically significant improvements in body image concerns Qualitative data showed improvement in body image concerns, emotional benefits, improved relationship with others and improved self-acceptance and awareness	Limited information about sources of potential bias related to participants’ personal characteristics, specific treatment and referral method	*Level 3* Non-randomized controlled cohort	Program called YBI program. Based on theory that yoga is a multidimensional tool that can promote embodiment and therefore improve body image which is particular issue with ED Group discussion aimed to enhance experience of embodiment through verbal processing and allowed peer-based discussion/support. Goal setting was used to encourage change Goal setting used Weekly themes based on improving body image (body awareness and appreciation, free movement, non-judgement, emotional connection, breathing, body-mind capabilities, resiliency and neutrality)
Fetterman [20] USA (dissertation only)	HIVE, stands for Healing, Integration, Vitality, and Experiential. 6 group sessions focussed on a theme Psychological Intervention: Aspects of psychoeducation, CBT, DBT and Disonnance interventions included in group discussion after yoga practice Homework prescribed each week focusing on journaling, daily meditation practice, and an at home 20 min yoga sequence. Journal assignments are related to theme for week Yoga Intervention: Yoga practice followed by a guided meditation	To describe HIVE program	No description regarding targeted participant group other than for people with an eating disorder No specific ED diagnosis or age range given				Program description only	*Dissertation only*	Weekly themes address core contributing theories of eating disorder symptomatology such as thin-ideal internalization, body appreciation and acceptance (body dissatisfaction), tolerance of negative affect, perfectionism, objectified body consciousness, poor interoceptive awareness, and low self-esteem Homework considered significant as a mechanism for change Group used to promote interpersonal learning and group cohesion
Clarke [15] USA (dissertation only)	Finding OM yoga program 10 week program including Yoga intervention: 1 h group yoga class in the Baptiste Power Vinyasa style using poses recommended to alleviate depression, anxiety and obesity. Class began and ended with guided meditation specific to BED-related issues such as distress tolerance, self image and binge/food related behaviour Psychological Intervention: 30 min psychoeducational discussion group	To assess whether the Finding Om yoga program, either with or without a CBT-style discussion component, would reduce bingeing behavior in people with BED	Females 18 year + with BED diagnosis 13 participants started study 3 participants dropped out 6 participants assigned to no discussion group (3 dropped out) 7 participants assigned to discussion group	Randomly assigned experimental design study with pre- post data	EDE-Q-I A yoga log (recording time and duration of yoga practice) Yoga Feelings Checklist Five factor mindfulness questionnaire (FFMQ) Body Responsiveness Questionnaire (BRQ)	Report of Binge eating episodes dropped significantly from 4.75 to 1.95 per week Significant reduction in scores on the EDE-Q-I. Significant increases in body awareness, body responsiveness and various facets of mindfulness were seen on the BRQ and FFMQ. There were no significant differences in results between the discussion group versus no-discussion group conditions, but anecdotal response indicated a perceived benefit of participation in the discussion group	Small sample size, imbalance of conditions and lack of a control group	*Dissertation only*	Yoga log was developed to replicate a food log used in BED treatment Yoga raises body awareness and responsiveness to bodily sensations [16], it was hypothesized that yoga practice would additionally help reduce bingeing behaviors The intention of the discussion was to consolidate and advance members’ treatment gains by capitalizing on the factors inherent in group, such as feelings of universality and the sharing of information (Yalom 1995) Weekly themes were specifically related to BED- issues such as distress tolerance, self-image and binge/food related behaviour

### Stage 4: Charting the data

Data relating to the included studies were extracted according to the PRISMA-ScR checklist [42] and a data charting form was developed to extract results relevant to answering the research question. The following data were chartered: study characteristics (author, year, country, design, and outcome measures) study aim and study population, program characteristics including intervention descriptions (both yoga and psychological approaches), underpinning conceptual frameworks and rationale for the program, study findings (results and limitations) and research implications.

### Stage 5: Collating, summarising and reporting results

Findings from the data charting form were presented in extraction tables. These were discussed among the research team and a narrative synthesis of the results was developed to address the central research question.

## Results

**Fig. 1:**
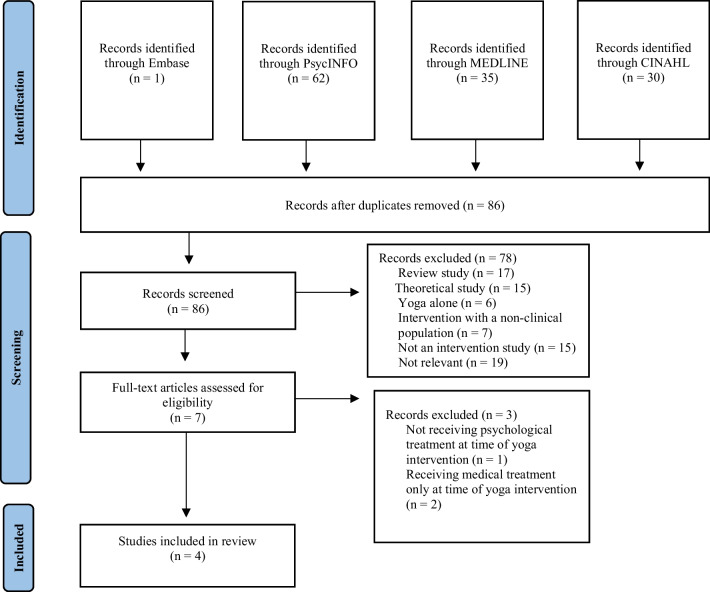
PRISMA workflow

The final search identified 86 relevant studies. Seventy-nine studies were excluded based on not addressing the research question, resulting in seven studies for full-text review (refer to Fig. 1). Following full-text review, three further studies were excluded. One was excluded since the yoga intervention occurred when participants were on the wait list for eating disorder treatment and therefore, participants were not receiving psychological treatment at the time of receiving the yoga intervention [5]. The other two studies were excluded since they examined yoga alongside medical rather than psychological treatment [7, 41].

Four studies remained. The first study examined an eight-week group program called Attunement in Mind, Body and Relationship (AMBR) that aimed to reduce symptomatology associated with AN and BN [8] for 29 adults in an outpatient program. The second study examined a different eight-week group program called Yoga Body Image (YBI) that aimed to reduce negative body image in 67 people diagnosed with an eating disorder in an outpatient program. The final two studies were unpublished doctoral dissertations. One described the rationale for the development of a six-week yoga program called the ‘HIVE’ program standing for Healthy, Integrative, Vitality and Experiential which aimed to provide skills and tools to build the mind–body connection and improve self-regulation and self-acceptance for people with an eating disorder [20]. The other examined the efficacy of a 10-week group program called Finding Om for 13 people (aged 19–47 with a mean age of 31.5 years) with a BED diagnosis in a community setting to reduce binge eating episodes [15]. All four studies were conducted in the USA, and all developed a novel group program that varied between 6 and 10 weeks in length. All programs were developed for people with an eating disorder which integrated yoga followed by a group discussion that was adapted from various psychological approaches. Both the yoga and the group discussion were specifically adapted for the population with the aim that it would be therapeutic and support eating disorder recovery. Outcomes from these studies included decreased symptomatology for AN, BN [8], BED [15] and improved body image [18].

In the first included study, the AMBR program [8] was evaluated. Pre-post change in Drive for Thinness (DT), Body Dissatisfaction (BD), and Bulimia (BU) were assessed via the Eating Disorder Inventory-2. Following the intervention, participants experienced significant reductions in DT and BD scores along with reductions in dieting, less fear of gaining weight, and decreased discontentment with the overall shape and size of their bodies. No significant differences in bulimia scores for those diagnosed with BN were found. The findings suggest incorporating yoga with existing evidence-based psychological treatments has the potential to relieve eating disorder symptoms. Individuals involved in the AMBR program experienced a reduction in eating disorder symptoms, but it was unclear what aspects of this program, if any, contributed to these changes since there was no control group. Other limitations include the small sample size, no follow-up analysis and the study design did not allow for comparisons between treatment components to examine how each contributed to positive outcomes separately and in combination.

The AMBR program [8] was based on the Attunement Theoretical Framework [9] for eating disorders, which was developed from an aggregate of meta-analyses and literature reviews. According to this framework, attunement is understood as the mutual influence and co-regulation between one’s internal system (thoughts, feelings, emotions) and external experiences (cultural and relational). Attunement is thought to occur when the relationship between one’s thoughts, feelings and emotions are aligned with their external experiences. This is similar to the concept of embodiment, as when internal experiences (thoughts, feelings and emotions) are attuned to external experiences (such as community, culture and relationships) positive embodiment is experienced. It is proposed that when the interaction between internal and external experiences is difficult to manage negative embodiment or disembodiment is experienced and may result in the adoption of eating disorder behaviours.

In the AMBR program, 50 min of body focussed yoga and 15 min of meditation were used in the first part of the session followed by group discussion with activities developed from CBT, DBT, and dissonance-based interventions. The authors describe selecting CBT, DBT and dissonance-based interventions given they are evidence-based therapies for eating disorders and use cognitive and behavioural exercises to improve thoughts, feelings, and emotions. Group discussion included mindfulness, emotion regulation and distress tolerance activities along with a focus on developing positive relationship skills and challenging society’s thin ideal represented in media. Weekly themes were based on the Attuned Theoretical Model and eating disorder recovery, starting with thoughts, feelings, and emotions (internal systems) followed by the impact of culture, community, and relationships (external system). Curriculum content (yoga practice and group discussion) was designed to align with the weekly theme with participants given related readings (selected poems and short readings) to review and were encouraged to reflect on these through journaling. The group component of this program was seen as an important aspect with members actively invited to contribute and support each other in their recovery.

In the second study, the Yoga Body Image (YBI) program aimed to reduce negative body image in people diagnosed with an eating disorder. Quantitative pre-post surveys based on the Body Shape Questionnaire [10] found a statistically significant improvement in both body image concerns, and less time spent thinking negatively about their body. Qualitative data suggested the yoga program improved participants’ self-acceptance, self-awareness, confidence, and emotional and physical strength. Results suggest it also promoted positive embodiment, possibly arising through verbal processing and peer-based discussion/support. Adversely, some elements of the qualitative data highlighted how, for some participants, physical and emotional challenges experienced throughout the program negatively impacted their engagement and contributed to self-judgement, vulnerability, and confrontation of uncomfortable feelings. A strength of this study design was the inclusion of both quantitative and qualitative outcome measures and the subsequent triangulation of data. Given there was no control group, it is unclear what aspects of this program, if any, contributed to these changes. Limitations of this study include the small number of participants and no follow-up analysis.

The authors of the YBI program reported the program was based on the theory that yoga is a multidimensional tool that can promote embodiment. Weekly sessions involved 45 min of therapeutic yoga that had aspects of hatha, vinyasa and viniyoga with postures, breathe techniques and guided imagery selected to align with the theme. This was followed by 45 min of group discussion that was aimed at encouraging participants to verbally process their experience by describing their sensations, emotions, and overall experience. Each session focussed on a different theme related to body image, such as body awareness and appreciation, free movement, non-judgement, emotional connection, breathing, body-mind capabilities, resiliency, and neutrality. Similar, to the AMBR program, YBI was designed to be delivered as a group intervention as the authors considered participant peer discussion and support to be an important therapeutic component in enhancing eating disorder recovery.

The third study an unpublished dissertation described the HIVE program [20] which aimed to provide skills and tools to build the mind–body connection and improve self-regulation and self-acceptance for people with an eating disorder. The HIVE program used yoga and guided meditation followed by group activities adapted from CBT, DBT and acceptance and commitment therapy (ACT). The authors describe selecting CBT, DBT and ACT as they are evidence-based theories for the treatment of eating disorders. Weekly themes were aimed at addressing core contributing theories of eating disorder symptomatology such as thin-ideal internalization, body appreciation and acceptance (body dissatisfaction), tolerance of negative affect, perfectionism, objectified body consciousness, poor interoceptive awareness, and low self-esteem. Yoga and group discussion were designed each week to align with these themes. Between sessions, meditation, journaling and an at home 20-min yoga sequence were provided for additional practice. Unfortunately, to our knowledge, this program was not further developed and or tested.

The final included study, another unpublished dissertation, evaluated the impact of ‘Finding OM’ [15] a program aimed to reduce BED symptoms. A randomly assigned experimental design was utilised with a control group receiving yoga only (*n* = 6) and the other receiving yoga followed by group discussion (*n* = 7). Three participants dropped out of the yoga only group and subsequently, the study was underpowered to sufficiently compare data between groups. Results indicated that for both groups there was a reduction in objective binge episodes, with binge episodes decreasing from 4.75 to 1.98 per week. A comparison of means between groups showed binge episodes in the yoga only group decreasing from 4.66 to 2.17 per week and yoga and discussion group 4.8 to 1.86 per week. However, the low number of participants and high dropout rate meant the study was underpowered to adequately compare data between groups. The authors concluded that a yoga-based intervention alongside psychological approaches might effectively support reducing binge eating episodes, but that larger controlled trials were required to support this finding.

The Finding OM program was developed based on the theory that exercise is a missing key component in the treatment of BED (as per Pendleton’s et al. 2005 theory). The program aimed to use yoga exercises to raise participants’ levels of mindfulness while moving, with the group discussion designed to improve participants’ distress tolerance. Weekly themes were specifically related to BED issues such as distress tolerance, self-image and binge/food-related behaviour with the yoga and group discussion designed to align with these. It was hypothesised that engagement in the program would improve the participants’ ability to regulate their emotions and this would subsequently lead to a decrease in binge eating episodes. Homework was set each week including journaling, along with asking participants to keep a yoga log which was developed as an adaptation to a similar ‘food log’. The program was designed as a group intervention as group factors such as feelings of universality and the sharing of information were seen to be therapeutic.

## Discussion

This review examined current research involving interventions incorporating yoga with psychological approaches that report how such approaches have been used to promote eating disorder recovery. Despite growing interest in the use of yoga as a complementary intervention for the treatment of eating disorders, there is a lack of research in this area, with only two published studies and two unpublished dissertations identified. All were conducted in the USA, and all developed a novel therapeutic group program designed specifically for people with an eating disorder that used a yoga practice in conjunction with various psychological approaches. All four studies used weekly themes related to ED recovery, with the yoga practice and group discussion designed to align with these themes. One unpublished dissertation [20] was a description of the yoga program only, but the other three studies trialled their programs in clinical populations with adults with eating disorders in the community [8, 15, 18]. Outcomes from these studies showed decreased symptomatology for AN, BN [8], BED [15] and improved body image [18]. However, considering these studies have significant methodological limitations such as small sample sizes, no control group or follow-up analysis, findings remain preliminary. All studies were completed in a community or outpatient setting so the findings of this review may not be able to be generalised to inpatient settings. Furthermore, no studies were with children or young people experiencing an eating disorder. Given the typical age of onset of eating disorders is adolescence, [32] and that it is common for services in the USA, UK, and Australia to offer an adjunctive yoga program alongside eating disorder treatment [4], it is apparent that the evidence base is lagging current practice approaches.

Despite all four studies providing a theoretical framework and rationale for why they incorporated yoga and psychological approaches, the differences between them suggest there are various perspectives on how yoga might complement or enhance psychological approaches. The most cited theoretical approach across the studies was the concept of ‘embodiment’ (used in the YBI program) which is, similar to attunement from the ‘Attuned Theoretical Framework’ (used in the AMBR program). Embodiment is commonly cited within the literature to explain how yoga can help with eating disorder recovery (through a mindful connection to self) [45] and aligns with the theoretical underpinnings of CBT-E; the ‘transdiagnostic model of eating disorders’ that calls for a multi-pronged approach to eating disorder treatment [22]. Given the theoretical alignment of yoga with evidence-based psychological approaches [25], the addition of yoga is considered to enhance embodiment, whilst also complementing the psychological approaches used. Nonetheless, none of the programs explicitly described how embodiment linked with the evidence-based psychological approaches utilised, or referred to eating disorder whole of body frameworks [21], suggesting the mechanism of change for how yoga enhances psychological therapy requires further examination. Given the lack of studies it remains unclear whether yoga being used in addition to a psychological approach enhances eating disorder recovery more than yoga used as a stand-alone intervention. In future research, when developing yoga programs researchers should identify and evaluate the proposed theoretical mechanisms of change for how the addition of yoga is thought to enhance eating disorder recovery. Further to this, the broader field could benefit from additional qualitative perspectives to understand these mechanisms from participants’ lived experiences [33].

Notably, the group design used by all four programs was considered an important factor in supporting therapeutic change. The group component was seen to offer opportunities for engaging in an interpersonal and social setting within which the individuals can engage in authentic discourse and begin self-exploration, providing social modelling and active practice in intra- and inter-personal integrative experiences [8]. Therapeutic group factors such as universality and the sharing of information were noted as important in supporting recovery [15]. On the other hand, qualitative findings from the YBI program [18], identified the challenges participants experienced in engaging with the group and how these contributed to uncomfortable feelings of self-judgement and vulnerability, highlighting the need to further understand these barriers.

Group programs are prominent within clinical guidelines for the treatment of eating disorders [22], demonstrating positive research outcomes, are cost-effective, and align with how yoga is typically taught in the community. Despite this, there are significant challenges when working with groups of people with eating disorder histories. Eating disorder symptomatology and presentations can differ significantly across a broad range of diagnoses and age ranges [22]. For example, a younger adolescent who has been recently diagnosed with BED will present differently to an older adult with longstanding AN and require different treatment approaches. Moreover, it is common for people with eating disorders to have perfectionistic personality traits, negative self-talk, and comparative thinking [47] which causes additional challenges when trying to develop critical therapeutic group factors (Yalom 1995). Given there were no studies identified that examined the use of yoga and psychological therapies in individual therapy for eating disorder treatment we are unable to compare individual versus group outcomes. Subsequently, future research could investigate whether yoga integrated with psychological approaches has better efficacy when implemented individually or within a group setting to answer this question.

### Research implications

Despite positive results for studies that trialled the novel group programs [8, 15, 18] this review identified limitations such as small sample sizes, no control groups, high rate of attrition, and no follow-up analyses. Subsequently, it remains unclear whether incorporating yoga with existing evidence-based psychological treatments has the potential to further relieve eating disorder symptoms and support recovery. It is suggested researchers consider integrating more rigorous research designs such as, clearly articulating theoretical concepts guiding program design and using multi-arm trials which can clarify which of the intervention components (yoga with or without psychological approaches) contributed to the outcomes. Trialling yoga programs that offer both individual and group approaches, with a range of ages, both in inpatient and outpatient settings would provide a further understanding of how yoga may enhance recovery at different stages across the lifespan. It is also suggested future research adopt participatory research approaches such as co-design methods [19], to enhance participant engagement and program acceptability, including consideration of diagnosis and age factors.

### Limitations

Although two unpublished dissertations were included in this study, there may be other grey literature publications that could have added more depth to the discussion. Further to this, given the lack of identified studies in this area conclusions are limited.

## Conclusion

This scoping review was the first to examine yoga alongside psychological approaches for the treatment of eating disorders. Findings highlight the paucity of available research with only four studies identified. The studies that piloted programs all identified promising results with a reduction of eating disorder symptomatology. However, these results remain tentative due to methodological limitations and the overall lack of available evidence. In the future, researchers are encouraged to clearly articulate the theoretical concepts that underpin their yoga programs and focus on adequately powered and designed trials, such as RCTs, to accurately compare treatment effects between interventions combining yoga with psychological interventions and standard psychological treatment. Qualitative enquiry is also recommended to provide further insights regarding what makes interventions successful. Current evidence suggests further guidance and pragmatic recommendations to guide researchers and clinicians alike are required; ultimately improving outcomes for people experiencing an eating disorder across the lifespan.

## Data Availability

Not applicable. None required as this study is a scoping review.

## References

[1] American Psychiatric Association (2013). Diagnostic and statistical manual of mental disorders.

[2] Australian Psychological Society (2018). Evidence-based psychological interventions in the treatment of mental disorders: a review of the literature.

[3] Arksey H, O'Malley L (2005). Scoping studies: towards a methodological framework. Int J Soc Res Methodol Theory Pract.

[4] Borden A, Cook-Cottone C (2020). Yoga and eating disorder prevention and treatment: a comprehensive review and meta-analysis. Eat Disord.

[5] Brennan MA, Whelton WJ, Sharpe D (2020). Benefits of yoga in the treatment of eating disorders: results of a randomized controlled trial. Eat Disord.

[6] Brosnan P, Nauphal M, Tompson MC (2021). Acceptability and feasibility of the online delivery of hatha yoga: a systematic review of the literature. Complement Ther Med.

[7] Carei TR, Fyfe-Johnson AL, Breuner CC, Brown MA (2010). Randomized controlled clinical trial of yoga in the treatment of eating disorders. J Adolesc Health.

[8] Cook-Cottone C, Beck M, Kane L (2008). Manualized-group treatment of eating disorders Attunement in mind, body, and relationship (AMBR). J Spec Group Work.

[9] Cook-Cottone C (2015). Incorporating positive body image into the treatment of eating disorders: a model for attunement and mindful self-care. Body Image.

[10] Cooper PJ, Taylor MJ, Cooper Z, Fairbum CG (1987). The development and validation of the Body Shape Questionnaire. Int J Eat Disord.

[11] Cox AE, McMahon AK (2019). Exploring changes in mindfulness and body appreciation during yoga participation. Body Image.

[12] Cox AE, Tylka TL (2020). A conceptual model describing mechanisms for how yoga practice may support positive embodiment. Eat Disord.

[13] Cramer H, Lauche R, Haller H, Steckhan N, Michalsen A, Dobos G (2014). Effects of yoga on cardiovascular disease risk factors: a systematic review and meta-analysis. Int J Cardiol.

[14] Cramer H, Lauche R, Anheyer D, Pilkington K, de Manincor M, Dobos G, Ward L (2018). Yoga for anxiety: a systematic review and meta-analysis of randomized controlled trials. Depress Anxiety.

[15] Clarke DP. Assessing finding Om: A yoga and discussion-based treatment for binge eating disorder. Doctoral dissertation, The University of the Rockies; 2008.

[16] Daubenmier JJ (2005). The relationship of yoga, body awareness, and body responsiveness to self-objectification and disordered eating. Psychol Women Q.

[17] Desikachar TKV (1995). The heart of yoga: developing a personal practice.

[18] Diers L, Rydell SA, Watts A, Neumark-Sztainer D (2020). A yoga-based therapy program designed to improve body image among an outpatient eating disordered population: program description and results from a mixed-methods pilot study. Eat Disord.

[19] Dimopoulos-Bick TL, O'Connor C, Montgomery J, Szanto T, Fisher M, Sutherland V (2019). “Anyone can co-design?”: a case study synthesis of six experience-based co-design (EBCD) projects for healthcare systems improvement in New South Wales, Australia. Patient Exp J.

[20] Fetterman JS (2017). HIVE: the proposal of a yoga-mindfulness program for eating disorders.

[21] Hay P (2020). Current approach to eating disorders: a clinical update. Intern Med J.

[22] Hay P, Chinn D, Forbes D, Madden S, Newton R, Sugenor L, Touyz S, Ward W, Royal Australian and New Zealand College of Psychiatrists (2014). Royal Australian and New Zealand College of Psychiatrists clinical practice guidelines for the treatment of eating disorders. Aust N Z J Psychiatry.

[23] Hay PJ, Mond J, Buttner P, Darby A (2008). Eating disorder behaviors are increasing: findings from two sequential community surveys in South Australia. PLoS ONE.

[24] Hilbert A, Hoek HW, Schmidt R (2017). Evidence-based clinical guidelines for eating disorders: international comparison. Curr Opin Psychiatry.

[25] Khalsa MK, Greiner-Ferris JM, Hofmann SG, Khalsa SB (2015). Yoga- enhanced cognitive behavioural therapy (Y-CBT) for anxiety management: a pilot q study. Clin Psychol Psychother.

[26] Khalsa SS, Portnoff LC, McCurdy-McKinnon D, Feusner JD (2017). What happens after treatment? A systematic review of relapse, remission, and recovery in anorexia nervosa. J Eat Disord.

[27] Klein J, Cook-Cottone C (2013). The effects of yoga on eating disorder symptoms and correlates: a review. Int J Yoga Therapy.

[28] Klump KL, Suisman JL, Burt SA, McGue M, Iacono WG (2009). Genetic and environmental influences on disordered eating: an adoption study. J Abnorm Psychol.

[29] Kramer R, Cuccolo K (2020). Yoga practice in a college sample: associated changes in eating disorder, body image, and related factors over time. Eat Disord.

[30] Lauche R, Sibbritt D, Ostermann T, Fuller NR, Adams J, Cramer H (2017). Associations between yoga/meditation use, body satisfaction, and weight management methods: results of a national cross-sectional survey of 8009 Australian women. Nutrition.

[31] Levac D, Colquhoun H, O'Brien KK (2010). Scoping studies: advancing the methodology. Implement Sci.

[32] Levine MP, Smolak L, Hayden H (1994). The relation of sociocultural factors to eating attitudes and behaviors among middle school girls. J Early Adolesc.

[33] Margison FR, Barkham M, Evans C, McGrath G, Clark JM, Audin K, Connell J (2000). Measurement and psychotherapy. Evidence-based practice and practice-based evidence. Br J Psychiatry J Ment Sci.

[34] McGowan J, Straus S, Moher D, Langlois EV, O'Brien KK, Horsley T (2020). Reporting scoping reviews—PRISMA ScR extension. J Clin Epidemiol.

[35] Munn Z, Peters MD, Stern C, Tufanaru C, McArthur A, Aromataris E (2018). Systematic review or scoping review? Guidance for authors when choosing between a systematic or scoping review approach. BMC Med Res Methodol.

[36] Neukirch N, Reid S, Shires A (2019). Yoga for PTSD and the role of interoceptive awareness: a preliminary mixed-methods case series study. Eur J Trauma Dissoc.

[37] Neumark-Sztainer D, Eisenberg ME, Wall M, Loth KA (2011). Yoga and Pilate associations with body image and disordered-eating behaviors in a population-based sample of young adults. Int J Eat Disord.

[38] Neumark-Sztainer D, Watts AW, Rydell S (2018). Yoga and body image: how do young adults practicing yoga describe its impact on their body image?. Body Image.

[39] National Institute for Health and Care Excellence (NICE). Eating disorders: recognition and treatment Full guideline. 2017. https://www.nice.org.uk/guidance/ng69/evidence/full-guideline-pdf-161214767896 28654225

[40] O'Shea M, Capon H, Evans S, Agrawal J, Melvin G, O'Brien J, McIver S (2022). Integration of hatha yoga and evidence-based psychological treatments for common mental disorders: an evidence map. J Clin Psychol.

[41] Pacanowski CR, Diers L, Crosby RD, Neumark-Sztainer D (2017). Yoga in the treatment of eating disorders within a residential program: a randomized controlled trial. Eat Disord.

[42] Page MJ, McKenzie JE, Bossuyt PM, Boutron I, Hoffmann TC, Mulrow CD, Shamseer L, Tetzlaff JM, Akl EA, Brennan SE, Chou R, Glanville J, Grimshaw JM, Hróbjartsson A, Lalu MM, Li T, Loder EW, Mayo-Wilson E, McDonald S, McGuinness LA (2021). The PRISMA 2020 statement: an updated guideline for reporting systematic reviews. BMJ.

[43] Perey I, Cook-Cottone C (2020). Eating disorders, embodiment, and yoga: a conceptual overview. Eat Disord.

[44] Peters M, Marnie C, Tricco AC, Pollock D, Munn Z, Alexander L, McInerney P, Godfrey CM, Khalil H (2020). Updated methodological guidance for the conduct of scoping reviews. JBI Evid Synth.

[45] Piran N, Neumark-Sztainer D (2020). Yoga and the experience of embodiment: a discussion of possible links. Eat Disord.

[46] Piran N, Teall T. The developmental theory of embodiment. Preventing eating-related and weight-related disorders: collaborative research, advocacy, and policy change, 169–198; 2012.

[47] Radunz M, Keegan E, Osenk I, Wade TD (2020). Relationship between eating disorder duration and treatment outcome: systematic review and meta- analysis. Int J Eat Disord.

[48] Rivest-Gadbois E, Boudrias MH (2019). What are the known effects of yoga on the brain in relation to motor performances, body awareness and pain? A narrative review. Complement Ther Med.

[49] Tricco AC, Lillie E, Zarin W, O'Brien KK, Colquhoun H, Levac D (2018). PRISMA extension for scoping reviews (PRISMA-ScR): checklist and explanation. Ann Intern Med.

[50] Wang WL, Chen KH, Pan YC, Yang SN, Chan YY (2020). The effect of yoga on sleep quality and insomnia in women with sleep problems: a systematic review and meta-analysis. BMC Psychiatry.

[51] World Health Organization (2020). International statistical classification of diseases and related health problems, 11th revision (ICD-11).

